# Complete genome assembly of *Enterococcus faecalis* strain HL1, isolated from an infant fecal sample

**DOI:** 10.1128/MRA.00558-23

**Published:** 2023-10-31

**Authors:** Kyoung Su Kim, Gun-Hyeong Lee, Hyun Ryul Bak, Yoon Mee Park, Seung Hwa Lee, Soo-Jong Hong, Dong-Woo Lee

**Affiliations:** 1 Department of Biotechnology, Yonsei University, Seoul, South Korea; 2 Department of Pediatrics, Childhood Asthma Atopy Center, Humidifier Disinfectant Health Center, University of Ulsan College of Medicine, Seoul, South Korea; University of Maryland School of Medicine, Baltimore, Maryland, USA

**Keywords:** genome assembly, *Enterococcus faecalis* HL1

## Abstract

We present the complete genome sequence of *Enterococcus faecalis* strain HL1, isolated from infant feces. *E. faecalis* gains significant attention for its therapeutic potential. The genome of *E. faecalis* HL1 consists of a 2.7 Mb circular chromosome with no plasmids, and it contains a total of 2,546 predicted coding genes.

## ANNOUNCEMENT

Enterococci, a type of lactic acid bacteria, are commonly found in the human intestinal tract, as well as in human female genital tract and oral cavity (1). *Enterococcus* species are round cocci that occur singly, in pairs, or as short chains. These gram-positive facultative bacteria can grow in aerobic and anaerobic conditions (2, 3). They exhibit a growth temperature range of 10°C to 45°C and can survive at 60°C for 30 min (4). The NCBI Taxonomy Browser (https://www.ncbi.nlm.nih.gov/Taxonomy/ Browser/wwwtax.cgi) lists 71 enterococcal species, including *E. faecalis*. Recent studies have highlighted the potential therapeutic uses of *E. faecalis* in gut immunity (5), reducing *Candida albicans* virulence (6, 7), and as an antinematodal agent (8). However, *E. faecalis* is also known for causing antibiotic-resistant infections in humans (9, 10).

The American Type Culture Collection offers 104 commercially available isolated *E. faecalis* strains (https://www.atcc.org/microbe-products). In this study, we obtained the whole genome of *E. faecalis* HL1, which was isolated from an infant fecal sample (0.1 g/mL in PBS) following four 10-fold serial dilutions. A 100 µL sample of the diluted solution was cultured on blood-glucose-liver agar plates supplemented with oxgall and gentamicin (11) at 37°C for 7 days. Strain HL1 has been deposited at at Korean Collection for Type Cultures (KCTC), under the accession number KCTC 15339BP.

Genomic DNA was extracted from the HL1 strain, which was anaerobically cultured in MRS medium at 37°C for 15 h, using the Wizard Genomic DNA Purification Kit (Promega), following the manufacturer’s instructions. Subsequently, a genomic DNA library was constructed using the (3.0) PacBio Microbial Library kit. Quality control of reads was conducted with a minimum accuracy of 0.99 using circular consensus sequencing analysis with the default parameters of SMRT Tools v12. Long-read sequencing was performed on the PacBio Sequel II platform (Pacific Biosciences, CA, USA), generating raw HiFi reads (83,248 HiFi reads; 771,697,404 bp HiFi read bases; HiFi N_50_, 10,360 bp; average read length: 9,269 bp; average read quality: Q30). *De novo* assembly was carried out using the SMRT Link v12.0.0.176214 with default parameters unless otherwise specified. The sequencing protocol achieved a mean coverage of 286× for the genome. The assembly produced a complete sequence of 2,695,805 bp, represented by a single circular contig. This sequence includes the origin of replication, has a GC content of 38%, and contains no plasmids. As a result, we concluded that it is the complete sequence. Gene prediction and annotation were performed using the NCBI Prokaryotic Genome Annotation Pipeline v6.5 with default parameters unless otherwise specified (12). The genome was predicted to contain 2,546 genes, including 2,472 coding DNA sequences, 14 pseudogenes, 74 RNA genes, 4/4/4 (5S/16S/23S) rRNAs, 58 tRNAs, and four non-coding RNAs.

## Data Availability

The whole-genome sequence of *E. faecalis* strain HL1 has been deposited in the NCBI database under the GenBank and BioProject accession numbers CP124778 and PRJNA964481, respectively. The raw sequencing data can be accessed under the BioSample and SRA accession numbers SAMN35302205 and SRR24683254.
